# Irisin and Incretin Hormones: Similarities, Differences, and Implications in Type 2 Diabetes and Obesity

**DOI:** 10.3390/biom11020286

**Published:** 2021-02-15

**Authors:** Nicola Marrano, Giuseppina Biondi, Anna Borrelli, Angelo Cignarelli, Sebastio Perrini, Luigi Laviola, Francesco Giorgino, Annalisa Natalicchio

**Affiliations:** Department of Emergency and Organ Transplantation, Section of Internal Medicine, Endocrinology, Andrology and Metabolic Diseases, University of Bari Aldo Moro, I-70124 Bari, Italy; nicola.marrano@uniba.it (N.M.); giuseppina.biondi2@gmail.com (G.B.); a.borrelli93@gmail.com (A.B.); angelo.cignarelli@gmail.com (A.C.); sebastio.perrini@uniba.it (S.P.); luigi.laviola@uniba.it (L.L.); annalisa.natalicchio@uniba.it (A.N.)

**Keywords:** irisin, incretin, GLP-1, pancreatic islets, beta-cells, type 2 diabetes

## Abstract

Incretins are gut hormones that potentiate glucose-stimulated insulin secretion (GSIS) after meals. Glucagon-like peptide-1 (GLP-1) is the most investigated incretin hormone, synthesized mainly by L cells in the lower gut tract. GLP-1 promotes β-cell function and survival and exerts beneficial effects in different organs and tissues. Irisin, a myokine released in response to a high-fat diet and exercise, enhances GSIS. Similar to GLP-1, irisin augments insulin biosynthesis and promotes accrual of β-cell functional mass. In addition, irisin and GLP-1 share comparable pleiotropic effects and activate similar intracellular pathways. The insulinotropic and extra-pancreatic effects of GLP-1 are reduced in type 2 diabetes (T2D) patients but preserved at pharmacological doses. GLP-1 receptor agonists (GLP-1RAs) are therefore among the most widely used antidiabetes drugs, also considered for their cardiovascular benefits and ability to promote weight loss. Irisin levels are lower in T2D patients, and in diabetic and/or obese animal models irisin administration improves glycemic control and promotes weight loss. Interestingly, recent evidence suggests that both GLP-1 and irisin are also synthesized within the pancreatic islets, in α- and β-cells, respectively. This review aims to describe the similarities between GLP-1 and irisin and to propose a new potential axis–involving the gut, muscle, and endocrine pancreas that controls energy homeostasis.

## 1. Introduction

Incretins are gut hormones that potentiate insulin secretion after meal ingestion in a glucose-dependent manner. In particular, the “incretin effect” refers to the ability of oral glucose to elicit a greater insulin secretory response than does intravenous glucose, despite inducing similar levels of glycaemia [1]. The two best-studied incretins, glucose-dependent insulinotropic polypeptide (GIP) and glucagon-like peptide-1 (GLP-1), exert insulinotropic actions through distinct G-protein-coupled receptors (GIP-R and GLP-1R respectively) that are highly expressed on islet β-cells but also on α- and δ-cells, as well as in nonislet cells (e.g., heart, gastrointestinal tract, kidney, and several regions of the central nervous system [CNS]; GIP-R is also expressed in bone and adipose tissue, while the presence of GLP-1R in human adipose tissue, liver, or skeletal muscle is equivocal) [2,3]. Increasing evidence suggests that GLP-1 is also produced by the pancreatic α-cells and can therefore be considered a new pancreatic islet hormone exerting insulinotropic and glucagonostatic effects locally via paracrine and/or autocrine actions [4].

On the other hand, irisin is a myokine firstly described in 2012 by Boström et al. [5] as a peroxisome proliferator-activated receptor gamma coactivator 1-α (PGC1-α)-dependent myokine, secreted following physical activity and able to drive brown fat-like development of white adipose tissue and thermogenesis. Many studies have explored the pleiotropic properties of irisin, demonstrating its pivotal role in the regulation of energy metabolism, by acting on several tissues and intervening in numerous biochemical pathways. Recently, it has been proposed that irisin could exert its effects via αV integrin receptors [6]. Although skeletal muscle accounts for approximatively 72% of the total amount of irisin in the circulation [5], several studies suggest that irisin can also be produced by the pancreatic islets [7,8,9], thus emerging as a new potential intra-islet hormone.

Interestingly, irisin and GLP-1 show comparable pancreatic and pleiotropic effects and activate similar intracellular pathways.

In light of this evidence, the current review aims to describe the similarities between GLP-1 and irisin and to propose a new potential axis—involving the gut, muscle, and endocrine pancreas—relevant for the control of energy homeostasis.

## 2. GLP-1 and Irisin Synthesis, Secretion, and Mechanism of Action: So Different but so Similar

**Figure 1:**
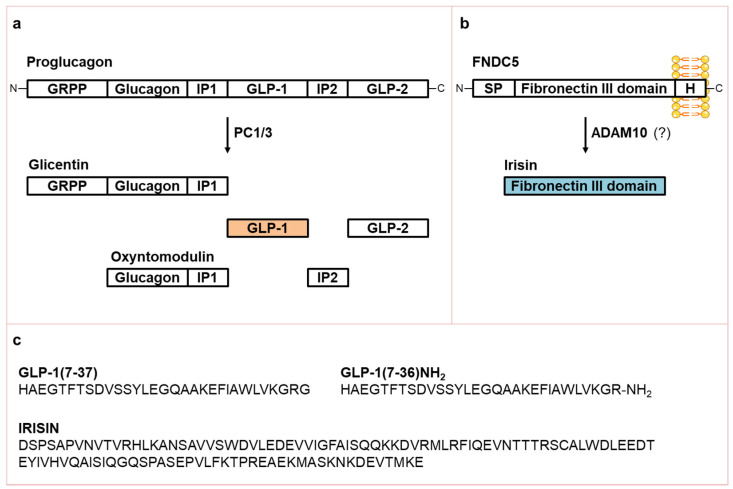
GLP-1 and irisin synthesis and aminoacid sequences. (**a**) Schematic representation of post-translational proglucagon processing by PC1/3; (**b**) Schematic representation of FNDC5 structure and cleavage to yield irisin; (**c**) aminoacid sequences of GLP-1(7–37), GLP-1(7–36)NH_2_, and irisin. ADAM10, A Disintegrin And Metalloproteinase domain-containing protein 10; C, C-terminal; FNDC5, fibronectin type III domain-containing protein 5; H, hydrophobic domain; GRPP, glicentin-related pancreatic polypeptide; GLP, glucagon-like peptide; IP, intervening peptide; N, N-terminal; PC1/3, prohormone convertase 1/3; SP, signal peptide.

GLP-1 is synthesized and secreted from enteroendocrine L cells scattered throughout the small and large intestine, following the post-translational processing of proglucagon by prohormone convertase 1/3 (PC1/3) (Figure 1a).

**Figure 2:**
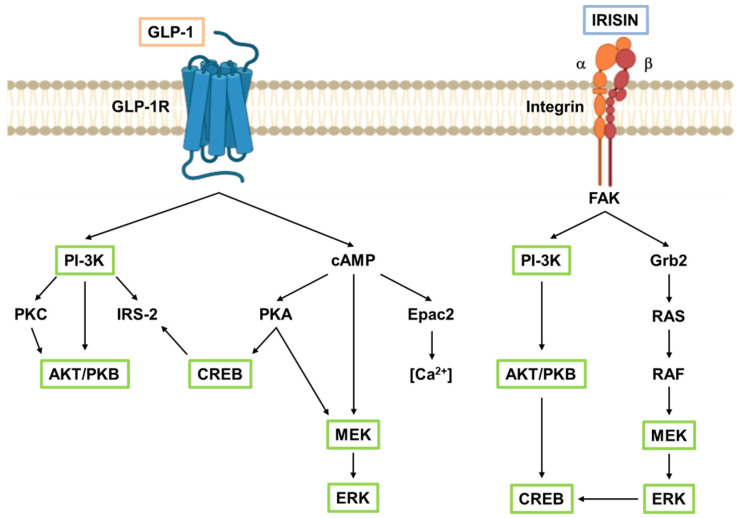
Main GLP-1R and integrin signaling pathways. GLP-1R and integrins, although structurally very different, share numerous intracellular signaling mediators (green boxes), ultimately leading to cell growth and survival. AKT/PKB, protein kinase B; cAMP, cyclic adenosine monophosphate; CREB, cAMP response element-binding protein; Epac2, exchange protein activated by cAMP 2; ERK, extracellular signal-regulated kinases; FAK, focal adhesion kinase; GLP-1, glucagon-like peptide-1; GLP-1R, glucagon-like peptide-1 receptor; Grb2, growth factor receptor-bound protein 2; IRS-2, insulin receptor substrate 2; MEK, mitogen-activated protein kinase; PI-3K, phospatidylinositol-3 kinase; PKA, protein kinase A; PKC, protein kinase C; RAF, rapidly accelerated fibrosarcoma; RAS, rat sarcoma.

In its biologically active form, GLP-1 is a 31-amino acid peptide designated as GLP-1(7-37). This peptide can be amidated at the C-terminal glycine residue to give rise to a second form of 30-amino acid peptide, GLP-1(7–36)NH_2_, that is biologically equipotent to GLP-1(7–37) and represents the major form in humans [10] (Figure 1c). The constant basal secretion of GLP-1 from enteroendocrine cells is rapidly augmented by the ingestion of luminal nutrients, mainly carbohydrates and fats and, to a lesser extent, proteins [11]. In addition, GLP-1 release can be stimulated by individual nutrients, including monosaccharides (glucose, galactose and fructose), fatty acids (FAs)–especially unsaturated FAs–and amino acids [3,12]. GLP-1 secretion occurs in a biphasic pattern starting with an early (within 10–15 min) phase that is followed by a longer (30–60 min) second phase [13]. In humans, the basal concentration of bioactive GLP-1 is 5–10 pmol/L and increases approximately 2–to 3-fold after a meal, depending on both the size and nutrient composition of the meal [3]. The half-life of GLP-1 in the circulation is less than 2 min due to rapid inactivation by the ubiquitous proteolytic enzyme dipeptidyl peptidase-4 (DPP-4) [14]. The GLP-1R belongs to the class B family of seven-transmembrane-spanning, heterotrimeric G-protein-coupled receptors. GLP-1R binding GLP-1 leads to the formation of cyclic adenosine monophosphate (cAMP) and the activation of phospatidylinositol-3 kinase (PI-3K), as well as related downstream pathways [3] (Figure 2).

Irisin is a 112-amino acid hormone (~12 KDa) that results from the proteolytic cleavage of the extracellular, N-terminal portion of the membrane protein fibronectin type III domain containing protein 5 (FNDC5; Figure 1b) [5]. Although the proteases responsible for FNDC5 cleavage are not yet fully identified, Yu et al. [15] have proposed that the metalloproteinase ADAM10 (A Disintegrin And Metalloproteinase domain-containing protein 10) could be the candidate enzyme responsible for irisin release. The amino acid sequences of irisin and GLP-1 are completely dissimilar (Figure 1c). The main stimulus to irisin expression and secretion is physical activity, varying in relation to intensity, type, time, and frequency [16]. A recent meta-analysis found that irisin concentration increased by ~15% immediately following an acute bout of exercise and identified fitness level as the single best predictor—being fit was associated with a nearly two-fold increase in post-exercise irisin concentration, compared with being unfit [17]. Considerable uncertainty remains regarding the veracity of methods used for the dosage of serum irisin [18]. Although Jedrychowski et al. [19] have indisputably detected and quantified circulating human irisin by tandem mass spectrometry, assessing that its concentration is ~3.6 ng/mL (~300 pmol/L) in sedentary humans and ~4.3 ng/mL (~358 pmol/L) in individuals undergoing aerobic interval training, currently available ELISA assays for quantifying circulating irisin levels still lack of quality and accuracy, and the measured values are very dissimilar between the different assays [18,20]. This may partially explain the discrepant data found by different research groups, especially in the setting of serum irisin measurements. As previously mentioned, irisin could exert its effects via αV integrin receptors, although further investigation is required to endorse this discovery [6]. α-integrins bind β-integrins to form obligate heterodimers that, upon ligand binding, usually trigger canonical signaling via phosphorylation of focal adhesion kinase (FAK), AKT/protein kinase B (PKB), and cAMP response element-binding protein (CREB) [21,22]. Indeed, GLP-1R and integrins, although structurally very different, share numerous intracellular signaling mediators (Figure 2, green boxes).

In addition to physical activity, the nutritional composition of the diet could also impact irisin secretion. We have previously demonstrated that a high-fat diet (HFD, 60% of energy from fat) causes a rapid and persistent increase of blood irisin concentrations, in healthy, wild-type mice [23]. In particular, irisin release from myotubes could also be influenced by the type of FA, as it appears to be stimulated by saturated, but not by monounsaturated, FAs [23]. Accordingly, Guilford et al. [24] showed that FNDC5 mRNA levels were increased in skeletal muscle and adipose tissue of HFD-fed mice. It has also been demonstrated in humans that the intake of saturated FAs with diet could increase irisin release [25]. Increased FNDC5/irisin may be a compensatory mechanism to offset HFD-induced weight gain and insulin resistance by increasing energy expenditure [24]. Conversely, de Macêdo et al. [26] showed that a high-carbohydrate diet and a HFD, but not a high-protein diet, reduced irisin protein expression in the soleus muscle of female mice. Importantly, in that study, circulating irisin levels were not measured, and this could explain the discrepancy with previous literature, since it has been demonstrated that regulation of irisin expression and its cleavage by FAs are two independent events [23]. Other studies failed to detect significant changes in circulating irisin levels [27] or found reduced irisin levels [28] in rats fed an HFD for 10 or 6 weeks, respectively. In those studies, however, irisin levels were measured only at the end of the nutritional intervention, whereas changes in irisin levels could be an acute response to nutrient ingestion, rather than a long-lasting effect. In obese subjects with metabolic syndrome, irisin concentrations were equally reduced after two weight loss diets differing in macronutrient composition; however, they were positively correlated with carbohydrate intake coming from cereals, pulses, fruit, and vegetables [29]. Similarly, irisin levels were directly associated with healthy diet types and patterns, particularly with increasing fruit consumption [30]. In contrast, Park et al. demonstrated that diet quality was not associated with irisin levels in humans [31]. Although limited and confounding data exist regarding the effect of the macronutrient composition of the diet on irisin levels—possibly due to differences in animal models or human cohorts, experimental diets, and methods and timing used for irisin measurement—meal composition does appear to affect irisin secretion, as is the case with GLP-1 (Table 1).

## 3. Incretins and Irisin in Type 2 Diabetes and Obesity: Two Sides of the Same Coin

It has been amply demonstrated that the “incretin effect” is typically reduced or even absent in people with impaired glucose tolerance or diabetes, and this contributes to defective insulin secretion and hyperglycaemia in patients with type 2 diabetes (T2D) [1]. As no consistent differences exist in the secretion of GIP [32] or GLP-1 [33] between individuals with and without T2D, the reduction of the “incretin effect” is the result of the total loss of the insulinotropic response to GIP and the reduced ability of GLP-1 to induce insulin secretion, likely due to a reduction of functional β-cell mass and receptor activity [1]. Because GLP-1, unlike GIP, retains substantial insulinotropic activity in diabetic patients, GLP-1R agonists (GLP-1RAs) and DPP-4 inhibitors are widely used as antidiabetic therapies. GLP-1RAs are particularly appreciated not only for their antihyperglycemic action but also for their cardiovascular benefits [34], as well as their ability to promote weight loss [35] and preserve/restore β-cell functional mass [36]. To date, exenatide, lixisenatide, liraglutide, dulaglutide, and semaglutide are the available GLP-1RAs.

Conversely, in a state of obesity, GIP levels are elevated during both fasting and after an oral glucose challenge, whereas GLP-1 levels are unchanged during fasting and reduced in response to an oral glucose challenge [37]. Overall, the “incretin effect” has been reported to be decreased in obesity, likely as a result of a reduced responsiveness to GIP or reduced contribution of GLP-1 to the insulin secretory response [38].

Likewise, despite the existence of conflicting results, most clinical studies, including the meta-analyses thereof, agree that circulating irisin levels are lower in patients with T2D [20,39,40,41]. In contrast, irisin levels appear to be higher in dysmetabolic states [42], such as obesity [43,44], possibly reflecting a condition of irisin resistance or a compensatory increase for the metabolic abnormalities and insulin resistance characteristic of these patients [44]. In addition, some studies have reported positive correlations between serum irisin levels and markers of adiposity (fat mass, waist circumference, waist-to-hip ratio) and between serum irisin levels and glucose and lipid homeostasis disturbances [44,45,46,47,48,49,50,51].

Numerous interventional studies in animal models of diabetes and/or obesity have shown that the exogenous administration of recombinant irisin can restore glucose and lipid homeostasis, thus exerting antidiabetic and antiobesity effects [5,52,53,54,55,56,57]. Taken together, these studies provide evidence regarding the involvement of irisin in the pathogenesis of metabolic diseases, supporting its possible therapeutic use.

Interestingly, metformin, the current first-line antidiabetic drug, is able to promote both the secretion of GLP-1, likely through indirect mechanisms [58] and the synthesis of irisin in rat pancreatic β-cells [9]. Finally, it has been demonstrated that obese T2D patients treated with exenatide for 12 weeks show significantly increased irisin levels, and this increase is correlated with an improvement in the metabolic profile [59], thus suggesting the existence of a possible interplay between irisin and incretin hormones.

## 4. Effects of GLP-1 and Irisin on Function and Survival of Pancreatic Islets

A deficit of β-cell functional mass is recognized as a necessary and early condition for the development of T2D. Thus, β-cell regeneration or the preservation of functional pancreatic islet integrity should be highly considered for T2D treatment and, possibly, cure [36].

**Figure 3:**
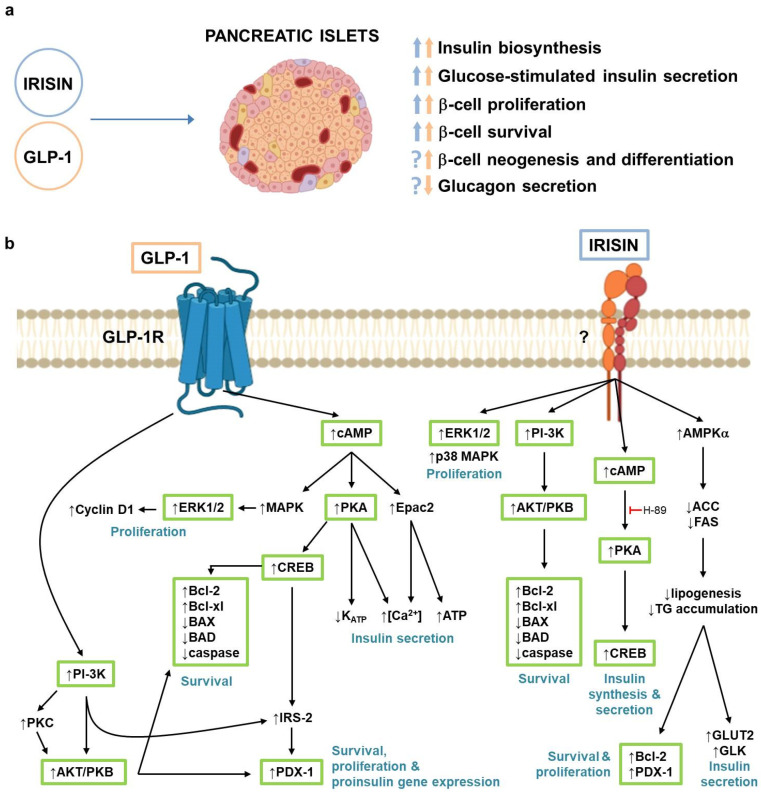
(**a**) GLP-1 and irisin effects in pancreatic islets. Blue arrows indicate irisin effects; orange arrows indicate GLP-1 effects; (**b**) Signaling pathways activated by GLP-1 and irisin in pancreatic β-cells. Green boxes indicate intracellular mediators activated by both GLP-1 and irisin. ACC, Acetyl-CoA carboxylase; AMPKα, adenosine monophosphate-activated protein kinase α; AKT/PKB, protein kinase B; BAD, Bcl-2 associated agonist of cell death; BAX, Bcl-2-like protein 4; Bcl-2, B-cell lymphoma-2; Bcl-xl, B-cell lymphoma-xl; cAMP, cyclic adenosine monophosphate; CREB, cAMP response element-binding protein; Epac2, exchange protein activated by cAMP 2; ERK1/2, extracellular signal-regulated kinases 1/2; FAS, GLK, glucokinase; GLP-1, glucagon-like peptide-1; GLP-1R, glucagon-like peptide-1 receptor; GLUT2, glucose transporter 2; IRS-2, insulin receptor substrate 2; MAPK, mitogen-activated protein kinase; PDX-1, pancreatic and duodenal homeobox 1; PI-3K, phospatidylinositol-3 kinase; PKA, protein kinase A; PKC, protein kinase C; TG, triglycerides.

In pancreatic β-cells, in addition to their known ability to induce glucose-stimulated insulin secretion (GSIS), GLP-1R activation results in increased glucose sensitivity, insulin biosynthesis, and β-cellular neogenesis and proliferation, while decreasing apoptosis [36] (Figure 3a). Indeed, numerous studies have demonstrated that GLP-1RAs are able to protect or restore human pancreatic β-cell function and mass, both in vitro and in vivo [36]. In addition, GLP-1 also inhibits glucagon secretion from pancreatic α-cells [60,61] (Figure 3a).

GLP-1 stimulates insulin secretion by intervening in nearly all steps involved in the exocytosis of insulin secretory granules (i.e., ATP synthesis, inhibition of potassium-ATP channels [K_ATP_], increase in intracellular Ca^2+^ levels) [3]. These events are mainly regulated by cAMP-protein kinase A (PKA) and cAMP-Epac2 (exchange protein directly activated by cAMP 2) signaling pathways [62] (Figure 3b). In particular, the cAMP-PKA pathway controls the inhibition of K_ATP_, and the cAMP-Epac2 pathway endorses the synthesis of ATP, while both signaling pathways promote the increase of intracellular Ca^2+^ levels [62]. Importantly, GLP-1 induces insulin secretion in a glucose-dependent manner, although the mechanisms are not yet fully understood. In addition, GLP-1 also increases proinsulin gene expression and insulin biosynthesis, through a mechanism involving cAMP/PKA-dependent and -independent signaling pathways [62,63].

The proliferative effects of GLP-1 require the activation of numerous intracellular mediators, particularly PI-3K, AKT/PKB, protein kinase C (PKC), insulin receptor substrate-2 (IRS-2), mitogen-activated protein kinase (MAPK), and extracellular signal-regulated kinases (ERK), leading to the upregulation of transcription factors, such as pancreatic and duodenal homeobox 1 (PDX-1) and CREB, and of the cell cycle regulator cyclin D1, all involved in the proliferation process (Figure 3b). These mechanisms have been demonstrated mostly in rodent models, since human β-cell proliferation in vitro is rarely observed. The protective effects of GLP-1 and GLP-1RAs against various toxic stimuli, including high-glucose, FAs, cytokines, and reactive oxygen species, have also been extensively investigated and involve multiple signaling pathways, most of which overlap with those inducing cell proliferation (i.e., PI-3K, AKT/PKB, PDX-1, CREB) and lead to the upregulation of antiapoptotic proteins, such as Bcl-2 and Bcl-xl, and downregulation of proapoptotic proteins, such as Bax, Bad, and caspases [62] (Figure 3b). We have previously demonstrated, in rat and mouse β-cell lines, as well as in human and murine pancreatic islets, that exendin-4 is able to prevent TNFα- [64] and palmitic acid-induced β-cell apoptosis [65] by reducing the ability of these stressful stimuli to activate proapoptotic effectors, such as the stress kinases c-jun N-terminal kinase (JNK) [64,65] and p38 MAPK [65], and the redox adaptor protein p66Shc [66]. Finally, GLP-1 is also able to preserve β-cell identity and promote the neogenesis of new β-cells starting from non-β-cell precursors, in both rodent models and human pancreatic ducts [62,67]. (For more detailed information concerning the mechanisms by which GLP-1 promotes β-cell function, survival, and regeneration, please see references [3,12,62]).

In pancreatic islets, irisin exerts effects that are very similar to those of GLP-1 (Figure 3a). We have previously demonstrated that recombinant irisin protects human and rodent β-cells and pancreatic islets from saturated FA-induced apoptosis by enhancing AKT/Bcl-2 antiapoptotic signaling [23] (Figure 3b). However, unlike GLP-1, irisin had no effects on FA-induced JNK, p38 MAPK, or p66Shc activation (unpublished data). Furthermore, irisin promoted proinsulin mRNA transcription and increased insulin content and secretion, in a PKA/CREB-dependent manner, as the PKA inhibitor H-89 abolished all these effects [23] (Figure 3b). The exact mechanism by which irisin induces insulin secretion is not yet known, however. Moreover, similar to GLP-1, irisin induced cAMP generation [23], suggesting the existence of a specific receptor.

In addition, irisin promoted β-cell proliferation through the activation of the ERK1/2 pathway [23]. Additionally, when administered in vivo, irisin improved GSIS and increased insulin content and β-cell mass and proliferation in healthy wild type mice, while only slightly reducing α-cell mass, although glucagon secretion was not investigated [23]. Importantly, both in vivo and in vitro, irisin enhanced insulin secretion only in a glucose-stimulated manner and not under low-glucose conditions, which can minimize the risk of hypoglycemia [23]. In accordance with these results from our group, Liu et al. [57] demonstrated that irisin significantly increased the proliferation of rat insulin secreting INS-1 cells via the ERK and p38 MAPK signaling pathways, protected the cells from high glucose-induced apoptosis by regulating the expression of pro- (Bax, Bad and caspases) and antiapoptotic (Bcl-2 and Bcl-xl) proteins, and improved pancreatic β-cell function in a T2D model in male rats. Furthermore, Zhang et al. [68] showed that irisin enhanced the expression of genes related to β-cell survival (e.g., PDX-1/Bcl-2) and function (e.g., GLUT2/glucokinase), thus reversing glucolipotoxicity-induced apoptosis and restoring insulin secretory ability in β-cells under glucolipotoxic conditions (Figure 3b). In particular, these effects were dependent on the activation of adenosine monophosphate-activated protein kinase (AMPK), the suppression of lipogenic gene expression (e.g., acetyl-CoA carboxylase), and therefore the reduced synthesis and intracellular accumulation of FAs/triglyceride [68]. Irisin also decreased the expression of proinflammatory genes [68].

Recently, it has been suggested that β-cells themselves could produce irisin, which could enhance β-cell survival and function by acting in autocrine-manner [9].

## 5. Extra-Pancreatic Effects of GLP-1 and Irisin: Beyond Glycemic Control

**Figure 4:**
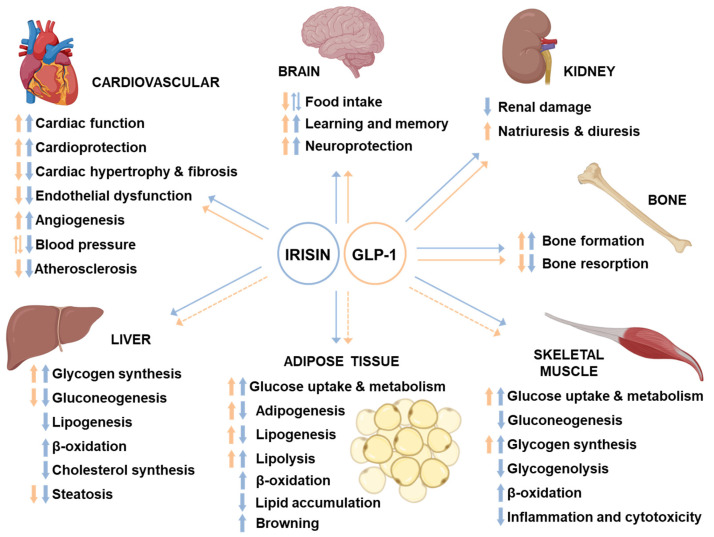
Pleiotropic effects of GLP-1 and irisin. Blue arrows indicate irisin effects; orange arrows indicate GLP-1 effects; dotted arrows indicate indirect effects.

The advent of GLP-1RAs represented a revolution in the treatment of T2D, because in addition to ensuring good glycemic control with a low risk of hypoglycemia and weight loss, they showed cardiovascular protective effects. These effects are in part dependent on the reduction of hyperglycemia but also involve direct effects of GLP-1 on several organs and tissues, such as the heart and vessels, CNS, and, to a lesser extent, kidney, liver, skeletal muscle, and adipose tissue. Likewise, irisin has shown similar pleiotropic effects (Figure 4).

### 5.1. Cardiovascular System

Although best known for their insulinotropic and weight-lowering actions, GLP-1 and GLP-1RAs also confer a series of beneficial effects on the cardiovascular system. These include the inhibition of cardiomyocyte apoptosis induced by different harmful stimuli [69,70,71,72,73,74], amelioration of endothelial dysfunction [75,76,77], and improvement of myocardial function and cardiac output in experimental models of cardiac injury or heart failure [69,78,79,80]. Indeed, GLP-1 reduces infarct size in the isolated perfused rat heart and in animal models of myocardial ischemia [81,82]. In addition, while some studies report increased heart rate (HR) and blood pressure (BP) in rodents upon acute or chronic GLP-1R agonism [83,84,85], other reports show decreased BP, especially in experimental models associated with the development of hypertension [86,87]. In humans, GLP-1RAs have shown moderate stimulatory effects on HR with reduced systolic BP in hypertensive and T2D individuals [88,89,90,91], and these effects are likely obtained through vasodilation and activation of nitric oxide-dependent mechanisms [12,91]. GLP-1RAs have also been demonstrated to exert a proangiogenic and antiapoptotic effect in human endothelial cells in vitro [92,93,94]. In addition, they have shown an antiatherogenic property both in animal models [95,96,97,98] and humans [99] (Figure 4). Finally, we have previously demonstrated that GLP-1RAs are also able to prevent palmitic acid-induced apoptosis [100] and H_2_O_2_-induced oxidative stress in human cardiac progenitor cells [101], which are essential for constant tissue repair and renewal in the adult heart.

Similarly, several recent reports have shown that irisin protects the heart against ischemia/reperfusion injury, improves cardiac function, and reduces infarct size, both in diabetic and nondiabetic rodents [102,103,104]. In addition, irisin improves myocardial performance (increased ejection fraction and reduced fibrosis) in *db/db* mice characterized by depression in cardiac function [105]. Interestingly, in a mouse model of acute myocardial infarction (MI), irisin administration significantly reduced infarct size and fibrosis, and alleviated MI-induced cardiac dysfunction and ventricular dilation [106]. Importantly, irisin administration significantly increased angiogenesis in the infarct border zone and decreased cardiomyocyte apoptosis but did not influence cardiomyocyte proliferation [106]. Accordingly, irisin has been shown to protect cardiomyocytes against apoptosis and dysfunction induced by different stressful stimuli, such as lipotoxicity [107], H_2_O_2_ [108], hypoxia-reoxygenation [109,110], and doxorubicin-induced oxidative stress [111], as well as alleviating angiotensin-II-induced cardiac hypertrophy and fibrosis [15,112,113] and pressure overload-induced cardiac hypertrophy [114]. The induction of autophagy has been proposed as a mechanism though which irisin exerts these beneficial effects [112,114,115], as block of the autophagic flux has been identified as a possible cause of cardiomyocyte apoptosis and cardiac hypertrophy. In addition, the activation of AKT [108,111,116] and AMPK [15,109,114] could represent key signaling steps in the cardio-protective effects of irisin. Moreover, irisin could also promote cardiac progenitor cell-induced myocardial repair and functional improvement in an infarcted heart [117]. Additionally, irisin was able to favor endothelium-dependent vasodilation and therefore to lower BP in obese mice and in the spontaneously hypertensive rat, through an AMPK-AKT-endothelial nitric oxide synthase (eNOS) dependent mechanism [118,119,120,121]. The BP-lowering effect of irisin has been examined in several studies. Peripheral administration of irisin reduced BP in both control and spontaneously hypertensive rats [122,123] through several different mechanisms, including induction of mesenteric arteries relaxation [122,124], activation of neurons controlling cardiac vagal tone [125], and augmentation of acetylcholine-mediated vasodilation [121].

Furthermore, in vitro studies conducted on human umbilical vein endothelial cells (HUVECs) or human microvascular endothelial cells (HMEC-1) have shown the irisin’s ability to exert proangiogenic effects through activation of the ERK-regulated proliferation signaling pathway [106,126,127,128], to prevent apoptosis induced by high fat, high glucose, or advanced glycation end products [128,129,130] and to counteract the impairment of angiogenesis by oxidized low-density lipoprotein (LDL) [116]. Interestingly, irisin could also improve endothelial function by increasing the number of endothelial progenitor cells (EPCs) in peripheral blood of diabetic mice and by improving the function of bone marrow-derived EPCs in diabetic mice via the PI3K-AKT-eNOS pathway [131]. Finally, irisin is able to inhibit atherosclerosis in apolipoprotein E-deficient mice by suppressing oxidized LDL-induced vascular inflammation and endothelial dysfunction [132] and promoting endothelial cell proliferation [127], likely through the AMPK-PI3K-AKT-eNOS signaling pathway [133] (Figure 4).

### 5.2. Central Nervous System

Both central and peripheral administration of GLP-1 and its analogs are able to lower body weight via centrally regulated inhibition of food intake in both healthy individuals and patients with T2D [134,135,136,137,138]. Of note, in rodents, intravenous infusion of liraglutide inhibits eating by directly promoting the expression of the hypothalamic anorexigenic genes cocaine- and amphetamine-regulated transcript (CART) and pro-opiomelanocortin (POMC) in the arcuate nucleus [139]. Importantly, liraglutide failed to suppress food intake in CNS-specific GLP-1R KO mice [140]. Consistent with this, in rats, central administration of the GLP-1R inhibitor exendin (9–39) was sufficient to attenuate the anorexigenic effect of peripherally administered liraglutide and exendin-4 [141], suggesting the importance of GLP-1R activation in the brain, and specifically in hypothalamic neurons, for the anorexigenic effects of GLP-1 and its analogs (Figure 4). Importantly, although GLP-1 and GLP-1RAs of relatively small molecular size (liraglutide, lixisenatide, and exendin-4) have all been demonstrated to cross the blood brain barrier (BBB) upon peripheral administration [142,143], the hypothalamus and hindbrain—both areas with incomplete BBB—appear to be the main targets of peripherally injected GLP-1RAs [12]. However, GLP-1RAs can also act on vagal afferents to transmit the signal to the hindbrain, which then projects to other key feeding areas in the hypothalamus [12]. Interestingly, GLP-1 is also synthesized by the hindbrain in a discrete set of neurons within the nucleus of the solitary tract [144,145,146], but little is known regarding the neurophysiology of these neurons and whether it is peripheral or central secretion of GLP-1 that regulates food intake.

On the other hand, the ability of irisin to modulate food intake in diabetic or obese mice/rats remains little investigated and the few existing results are conflicting [53,54]. We have recently demonstrated that daily intraperitoneal injection of irisin for 14 days in healthy C57BL/6 mice increases mRNA levels of the hypothalamic anorexigenic genes CART and POMC, similar to GLP-1, without affecting mRNA expression of the orexigenic genes agouti-related neuropeptide (AgRP), orexin, and promelanin-concentrating hormone (PMCH), and without inducing apparent changes in food intake [147]. Similarly, Butt et al. [148] showed that intraperitoneal irisin injection in goldfish increased expression of CART mRNA in the brain, without affecting AgRP mRNA expression, and induced a decrease in food intake, confirming that irisin may act as an anorexigenic factor in fish. In another study, intrahypothalamic injection of irisin for 24 h in rats augmented CART/POMC and reduced orexin mRNA levels, without affecting mRNA levels of the orexigenic neuropeptide Y (NPY) and AgRP, promoting a reduction in food intake [149]. Conversely, in another study in nonobese, nondiabetic rats, intracerebroventricular infusion of irisin for 7 days increased food intake and was associated with decreased POMC mRNA levels in the hypothalamus [150]. Considering these results, it is possible that the effects of irisin administration on food intake may be dependent upon the timing and/or route of administration (Figure 4). Importantly, like GLP-1, irisin can be locally expressed in the brain, since this peptide has been detected in the cerebrospinal fluid, and FNDC5 is known to be highly expressed in the glia (e.g., astrocytes and microglia) and neurons of various brain regions (e.g., cerebellar Purkinje cells, hypothalamus, hippocampus) [151,152]. Furthermore, peripheral irisin can also cross the BBB, although the mechanism of transport from the blood to the cerebrospinal fluid remains unknown [153,154].

In addition to affecting feeding behavior, central GLP-1R agonism has been demonstrated to have neuroprotective effects [155,156] and to improve several aspects of learning and memory [157]. The molecular mechanisms underlying the neuroprotective effects of central GLP-1R agonism are, at least in part, mediated by cAMP formation and subsequent PI-3K and ERK activation [12]. Impaired cerebral glucose metabolism and brain insulin resistance are main pathological features of Alzheimer disease (AD), and this has recently led researchers to designate AD as “diabetes of the brain” or “type 3 diabetes” [158]. Accordingly, GLP-1RAs have been tested and have shown encouraging protective actions in experimental models of AD, as well as in initial clinical trials [159,160,161,162] (Figure 4).

Similarly, several studies have demonstrated that irisin plays a developmental role in regulating the process of neuronal differentiation and maturation [152], induces the expression of neurotrophic factors, such as brain-derived neurotrophic factor (BDNF) [147,152], and could exert neuroprotective effects on neurodegenerative diseases, improving memory impairment and synaptic plasticity [152]. Interestingly, irisin improves learning and memory function, promotes neurogenesis, and prevents the neuronal damage caused by oxidative stress, thus representing a potential future target for ameliorating AD pathology and preventing AD onset [151,163] (Figure 4).

### 5.3. Kidney, Liver, Skeletal Muscle, Adipose Tissue, and Bone

The GLP-1R is expressed in the kidney and intravenous infusion of GLP-1 in animal models produces natriuretic and diuretic responses that are associated with increased glomerular filtration rate and inhibition of sodium reabsorption in the proximal tubule, thus exerting an antihypertensive effect [3]. The few studies in the literature that have examined the effects of irisin on the kidneys agree that this myokine is able to improve kidney dysfunction, as well as to reduce kidney injury and fibrosis caused by various stressful stimuli [164,165,166,167,168] (Figure 4).

It is not definitively clear if the effects of GLP-1 on liver, skeletal muscle, and adipose tissue depend on a direct effect or are the result of the overall amelioration in whole-body insulin sensitivity. Indeed, the presence of GLP-1R in these tissues is equivocal [2,3,12], although more recent studies have clearly identified that GLP-1R is expressed both in skeletal muscle [169,170] and adipocytes [12]. Accordingly, in skeletal muscle, GLP-1 increases glucose uptake, insulin-stimulated glucose metabolism, and glycogen synthesis [3]. In addition, GLP-1 also enhances glucose uptake and insulin-stimulated glucose metabolism in adipocytes [3]. Moreover, in human adipocytes, GLP-1 displays both lipolytic and lipogenic actions [171], as well as adipogenic effects [172]. Finally, it has been shown that GLP-1 inhibits hepatic glucose production and increases glycogen synthesis in hepatocytes [3], whereas exendin-4 improves insulin sensitivity and reverses hepatic steatosis in *ob/ob* mice [173,174]. Other studies, however, do not support a direct role for GLP-1R signaling in these tissues [175,176], and whether or not GLP-1R agonists can influence glucose disposal and insulin sensitivity independent of changes in insulin or glucagon remains unclear (Figure 4).

Conversely, numerous studies have confirmed the ability of irisin to directly act on liver, skeletal muscle, and adipose tissue. Indeed, irisin has been demonstrated to inhibit lipogenesis, cholesterol synthesis, and gluconeogenesis [55,177,178,179,180,181,182,183], activate FAs oxidation and glycogen synthesis [55,181,182,183], and reduce lipid accumulation, steatosis, and insulin resistance [178,180,181] in the liver. Finally, it has been shown that irisin is able to protect hepatocytes from injury caused by different harmful stimuli, such as methotrexate [184], ischemia-reperfusion [185,186], and glucose/lipid overload [183]. These effects suggest a lipid-lowering and hepatoprotective attitude of irisin. In addition, several papers have shown that irisin can act in skeletal muscle, promoting myogenesis, muscle hypertrophy [187] and metabolism, enhancing insulin sensitivity under basal [188] or lipotoxic [189,190,191] conditions, and protecting myocytes from high glucose- and FAs-induced cytotoxicity [188]. In particular, irisin improves glucose uptake [55,188,192,193,194] and inhibits gluconeogenesis and glycogenolysis [194], while increasing glycogen storage [188,194] and stimulating FAs oxidation [55,194,195]. The activation of AMPK appears to be a key signaling step for irisin action in the liver and skeletal muscle. In adipose tissue, it has been shown that irisin reduces adipogenic differentiation of preadipocytes [196,197] and promotes glucose uptake and glucose and lipid metabolism in mature adipocytes [197,198]. In addition, overexpression of FNDC5 in obese mice reduces the size of adipocytes in subcutaneous adipose tissue and stimulates lipolysis [53]. Adipocytes isolated from animals injected with irisin, or treated ex vivo, are therefore smaller and accumulate fewer lipids than do controls [53]. Furthermore, irisin treatment reduces lipid accumulation in human and mouse adipocytes [198,199] (Figure 4). According to Boström et al. [5], irisin also drives the browning of white adipose tissue by stimulating the expression of uncoupling protein-1 (UCP-1) and other known brown fat genes, while downregulating genes characteristic of white fat development, thus promoting thermogenesis and energy expenditure. In human adipocytes, the ability of irisin to induce browning is still debated: it is likely that only a small subpopulation of adipocytes that highly express “brite”-specific markers is responsible for the irisin effect [200]. In addition, Zhang et al. [196] suggested that the effects of irisin in humans are likely differentiation stage-dependent, as irisin induces browning in mature adipocytes but not in preadipocytes (Figure 4).

It has been reported that GLP-1 can promote bone formation and inhibit bone resorption in animal models, thus enhancing bone mineral density and quality [201] (Figure 4). These effects are of interest for T2D patients, as they are characterized by higher risk of bone fractures compared with healthy individuals [202], likely due to a reduction in bone turnover [203]. Promotion of GLP-1-induced bone formation may depend on the increase in the number of osteoblasts [204], in the expression of genes [205,206] and serum markers [205,207] related to bone formation, as well as, indirectly, on the reduction of glycemia [201]. All these effects are possibly mediated by MAPK [208] and Wnt [209,210] pathways, or c-fos transcription promotion [201]. On the other hand, GLP-1 inhibits bone resorption probably by reducing the number of osteoclasts [205] and promoting calcitonin secretion [201]. Unfortunately, the specific process and related molecular pathways by which GLP-1 acts on the bone are still not completely understood, and the relationship between GLP-1 and bone fractures is still under investigation [201]. Interestingly, irisin has also shown to exert anabolic effects on bone in mice [211]. Specifically, irisin promotes osteoblast differentiation [212], increases cortical bone mass, and makes bone more resistant and less susceptible to fractures [213] (Figure 4). As for GLP-1, also irisin effects could depend upon the MAPK pathway activation [214]. Additionally, in humans, irisin was inversely correlated with the incidence of bone fractures in postmenopausal osteoporotic women, as well as in patients with T2D, cardiovascular disease, and liver disease [215].

## 6. GIP and Irisin: Similarities and Differences Not to Be Underestimated

In this review we have focused on the similarities between irisin and GLP-1 because of the therapeutic potential of GLP-1, rather than GIP, in T2D. Nevertheless, there are similarities and differences also between irisin and GIP that should not be underestimated.

**Figure 5:**
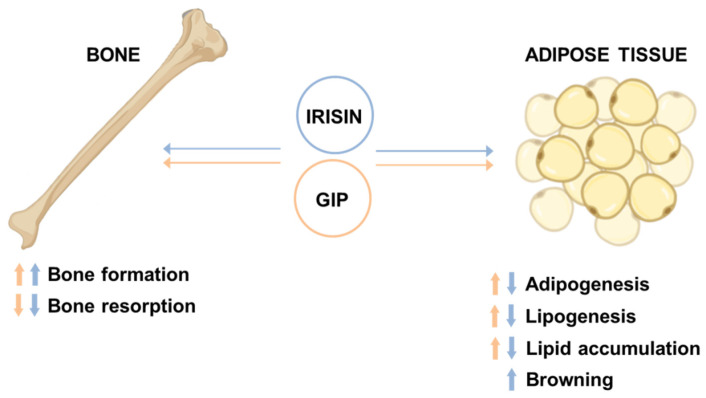
Glucose-dependent insulinotropic polypeptide (GIP) and irisin similarities and differences. Blue arrows indicate irisin effects; orange arrows indicate GIP effects.

GIP is released by intestinal K cells and, similar to GLP-1, is secreted in response to nutrient ingestion and activates GIP-R in pancreatic β-cells, enhancing meal-stimulated insulin secretion in a glucose-dependent manner and stimulating β-cell survival and, especially in rodents, proliferation [3]. In addition, GIP has similar effects to GLP-1 in the hypothalamic regulation of food intake [38,216]. Because improving glucose control with antidiabetic therapies appears to restore the insulinotropic effect of GIP in patients with T2D, growing evidence suggests that GLP-1R/GIP-R co-agonists could represent a promising strategy for treating T2D and obesity [217]. In particular, the overlapping of insulinotropic and body weight-lowering actions of both GLP-1 and GIP may have the potential to bolster their glucose-lowering and appetite-suppressing effects beyond what is observed with individual agents [217]. Importantly, GIP also targets bone similar to irisin, inhibiting bone resorption while promoting its formation, and thus exerting anabolic effects [3,218,219] (Figure 5).

Moreover, GIP also targets adipose tissue, stimulating adipogenesis, lipogenesis, and lipid storage in fat cells [3,217]. It has been suggested that the increase in lipid-buffering capacity of adipose tissue reduces lipid “spillover” and ectopic fat accumulation in tissues such as liver, skeletal muscle, heart, and pancreas, thus improving insulin sensitivity [220]. In contrast, as mentioned in the previous paragraph, the overall effects of irisin on adipose tissue lead to a reduction in fat mass and an increase in energy expenditure (Figure 5), which together may promote weight loss.

## 7. Conclusions

By definition, incretins are hormones with low basal plasma concentrations that are released after the ingestion of physiological nutrients to reach concentrations that augment the insulin secretory responses at a permissive degree of hyperglycaemia, although they are ineffective at low glucose concentrations [221]. According to this definition, irisin can be considered an incretin-like hormone, as its secretion appears to be also affected by meals and, once secreted, it enhances insulin secretion in a glucose-dependent manner. In addition, both the GLP-1 insulinotropic effects and irisin levels are defective in T2D, and their exogenous administration is able to improve glycemic control. Importantly, irisin and GLP-1 share numerous direct pancreatic effects (Figure 3), including the ability to induce insulin biosynthesis and glucose-dependent insulin secretion, as well as improving β-cell proliferation and survival. Albeit through different pathways, irisin and GLP-1 activate the same intracellular signaling proteins (e.g., AKT, CREB, ERK1/2; Figure 3b). Interestingly, irisin shows cardiovascular and anorexigenic effects similar to those of GLP-1, while demonstrating a more pronounced beneficial metabolic effect in insulin-dependent organs, such as liver, skeletal muscle, and adipose tissue (Figure 4). Of note, because GLP-1 and irisin signal through different receptors that nevertheless converge on very similar pathways (Figure 2 and Figure 3b), an additive effect of the two molecules cannot be excluded.

Additionally, like GIP, irisin promotes bone formation while, unlike GIP, it reduces lipid accumulation in adipose tissue (Figure 4 and Figure 5). Irisin could therefore broaden the beneficial effects of GLP-1RAs by restoring bone turnover and improving weight loss through the reduction of fat mass. Furthermore, recent evidence suggests that both GLP-1 and irisin are also synthesized within the pancreatic islets, lengthening the list of intra-islet hormones and paving the way for new scenarios in interorgan cross-talk.

In conclusion, due to its modalities of secretion and its pancreatic and extra-pancreatic effects, irisin could be considered an incretin-like hormone with an action similar to that of GLP-1, with the addition of an anabolic GIP-like effect at the bone level and a marked antilipogenic action. This evidence highlights the promising antidiabetic and antiobesity potential of irisin, possibly in combination therapeutic regimens with GLP-1RAs.

## Figures and Tables

**Table 1:** Effect of dietary nutrients on secretion of GLP-1 and irisin.

Nutrient	GLP-1 Secretion	Irisin Secretion
Carbohydrates	++	+
Lipids	++ ^1^	++ ^2^
Proteins	+	N/A

^1^ Especially unsaturated FAs, ^2^ Especially saturated FAs, N/A not available.

## Data Availability

Not applicable.
